# The Dutch Citizen's Understanding and Perception of the Actors Involved in the Netherlands' COVID‐19 Pandemic Response: A Focus Group Study During the First Pandemic Wave

**DOI:** 10.1111/hex.14170

**Published:** 2024-09-06

**Authors:** L. S. Kengne Kamga, A. C. G. Voordouw, M. C. De Vries, E. Belfroid, A. E. M. Brabers, J. D. De Jong, L. C. van Eck, M. P. G. Koopmans, A. Timen

**Affiliations:** ^1^ Centre for Infectious Disease Control, National Institute for Public Health and the Environment Bilthoven The Netherlands; ^2^ Athena Institute VU University Amsterdam Amsterdam The Netherlands; ^3^ Center for Infectious Disease Research, Diagnostics and Laboratory Surveillance, National Institute for Public Health and the Environment Bilthoven The Netherlands; ^4^ Netherlands Institute for Health Services Research Utrecht The Netherlands; ^5^ CAPHRI Maastricht University Maastricht The Netherlands; ^6^ Department of Viroscience Erasmus Medical Center Rotterdam The Netherlands

**Keywords:** COVID‐19, Dutch citizen involvement, focus group study, pandemic response actors, public health emergency

## Abstract

**Introduction:**

The COVID‐19 pandemic was a public health emergency (PHE) of unprecedented magnitude and impact. It provided the possibility to investigate the Dutch citizens' understanding and perception of the actors involved in the Dutch pandemic response as a PHE unfolded.

**Methods:**

Three focus groups (FGs) were held with 16 Dutch citizens in June 2020. Citizens were recruited using the Dutch Health Care Consumer Panel. During the FGs, participants were asked to fill in a table with actors they thought were involved in the management of the COVID‐19 pandemic. They also received information on actors involved in Dutch outbreak responses. Then, the actors named and omitted by the participants were discussed.

**Results:**

An analysis of the FGs suggests that the Dutch citizens participating in the study were not fully aware of the scope of actors involved in the Dutch COVID‐19 pandemic response. Some participants would have appreciated more information on the actors involved. This would help them have an informed opinion of the actors involved in the decision‐making process, and accept non‐pharmaceutical interventions implemented. Lastly, most participants recognised that they played a role in limiting the spread of the COVID‐19 pandemic. Yet, very few spontaneously mentioned themselves as actors within the COVID‐19 pandemic response.

**Conclusion:**

This study suggests that early in the COVID‐19 pandemic, the Dutch citizens participating in this study's FG did not have a complete understanding of the scope of actors involved in the Dutch COVID‐19 pandemic response, or the potential role of the citizen. Future research can build on these results to explore the citizen's perception of their role during PHEs of another origin, as well as other geographical and historical contexts.

**Patient or Public Contribution:**

The public participated in the focus groups and received a non‐expert report summarising the outcomes of the focus groups.

## 1 Introduction

Severe acute respiratory syndrome coronavirus 2 (SARS‐CoV‐2) was first reported in Wuhan in China's Hubei province in December 2019 [1]. It resulted in the COVID‐19 pandemic, a public health emergency (PHE) of unprecedented magnitude. The COVID‐19 pandemic and its consequences had an impact on all aspects of society [2, 3]. Hence, it required a multisectoral response including a variety of actors. Actors are subnational, national and international individuals or organisations that can make decisions or execute tasks. These can range from individual community leaders to the World Health Organization (WHO).

The actors responsible for the Dutch national response to large‐scale infectious disease outbreaks are described in the 2008 Public Health Act [4, 5]. In addition to the actors described there, a multitude of actors are involved in the implementation of (sub)national policies. The tasks of the actors involved in the pandemic response include developing and implementing policies related to control interventions, clinical management of patient cases and their contacts, testing and vaccinations. Normally, the response to infectious disease outbreaks is decentralised in the Netherlands. However, the SARS‐CoV‐2 virus was considered a serious threat to public health [6]. Hence, it was labelled a high‐priority notifiable disease by the Dutch government. This resulted in central and national response being led by the Ministry of Health, Welfare and Sports (MoHWS) [7, 8, 9] with the decision‐making power over the national legislations and regulations. It received epidemiological updates plus disease control and prevention advice from the National Institute for Public Health and the Environment (NIPHE). The Outbreak Management Team (OMT), an emergency organisation of experts chaired by the NIPHE, provided professional advice to responsible policymakers at the MoHWS. The OMT's advice was assessed for political and administrative feasibility by the Policy Advisory Committee (PAC).

The MoHWS tried to limit the spread of the SARS‐CoV‐2 virus by advising a range of non‐pharmaceutical interventions. The prime minister (PM) communicated the first set of non‐pharmaceutical interventions during a press conference on 1 March 2020 [6] focussing on hygiene measures. Thereafter, press conferences kept the public informed of the current epidemiological situation and non‐pharmaceutical interventions. A couple of weeks later, an extreme non‐pharmaceutical intervention was introduced, namely the ‘intelligent lockdown’. During the ‘intelligent lockdown’, Dutch citizens were urged to stay at home as much as possible and apply social distancing. Yet, their freedom of movement was not restricted [8, 10, 11]. However, the PM repeatedly stressed that the success of this approach depended on the Dutch citizens' ability to take responsibility, have self‐discipline and adhere to the interventions introduced [12, 13].

The WHO recognises the benefit of citizen participation during PHE preparedness and response (PHEPR). It advocates a *whole‐of‐society* approach, which encourages multisectoral collaboration whilst underlining the role of the community [14, 15]. The community is not only primarily affected by the pandemic but can also contribute to managing it [14, 15]. However, there is limited literature on citizen's own perspectives of PHEPR, or their potential role in preparedness and response.

Gaining insight into the citizen's perspective can provide valuable information on how to manage PHEs using a whole‐of‐society approach. The COVID‐19 pandemic provided the possibility to investigate Dutch citizens' perspectives on specific aspects of PHEPR as the pandemic evolved. The whole‐of‐society approach was also relevant during the COVID‐19 pandemic as a certain degree of citizen responsibility was publicly communicated by the PM. The citizens' compliance with the interventions was considered key to reducing the pace of the transmission of the virus, enabling the protection of vulnerable persons and ensuring access to healthcare.

It can be assumed that for citizens within communities to participate in PHEPR, they must first understand PHEPR. To assess Dutch citizen's understanding and perception of the actors involved in the COVID‐19 pandemic response, an exploratory, qualitative focus group (FG) study was conducted amongst Dutch citizens within the first 6 months of the start of the COVID‐19 pandemic—at a time when awareness of a PHE was high.

## 2 Methods

### 2.1 Context

This study was conducted within the context of the European Union Joint Action on Strengthening International Health Regulations & Preparedness in the EU (EU SHARP JA). The EU SHARP JA is a European collaborative action that aims to improve the implementation of the 2005 International Health Regulations (IHR) and EU Decision 1082/2013 on serious border threats to health [16]. One of the JA's priorities is multisectoral collaboration during preparedness and response planning.

**Figure 1:**
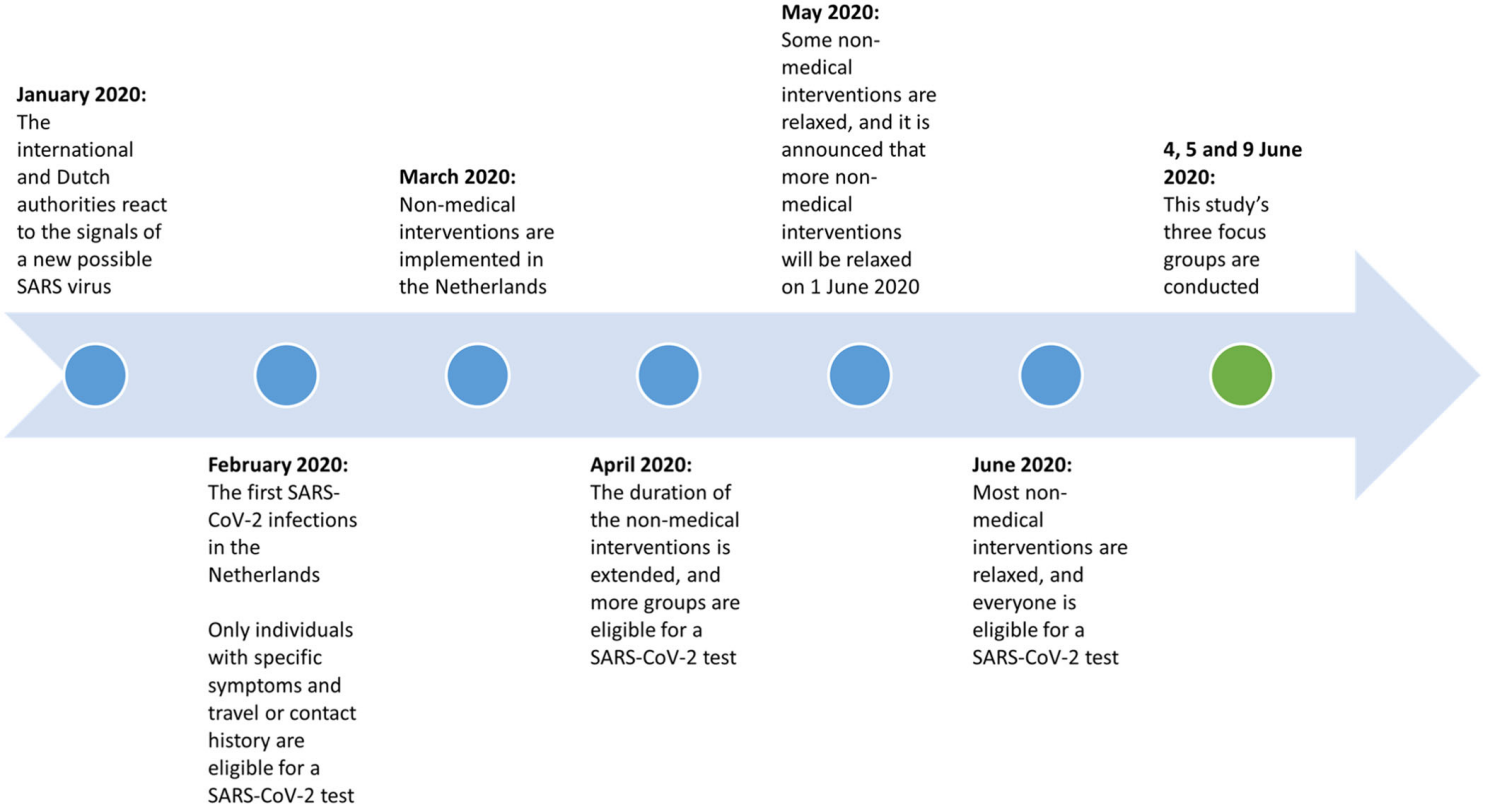
Key moments of the COVID‐19 pandemic in the Netherlands, January–June 2020.

Three FGs with Dutch citizens were planned on 4, 5 and 9 June 2020. At the time, the COVID‐19 pandemic was ongoing internationally for 6 months. The Dutch ‘intelligent lockdown’ implemented 3 months earlier had just been partly relaxed. Several key moments of the COVID‐19 pandemic in the Netherlands are shown in Figure 1.

Given the novelty of the situation and the lack of literature on the topic, an exploratory research approach was adopted to facilitate in‐depth and thoughtful discussions.

### 2.2 Recruitment

The participants were conveniently sampled using the Dutch Health Care Consumer Panel (DHCCP) [17]. The panel is managed by the Netherlands Institute for Health Services Research (Nivel). During the recruitment, a random sample of 1500 panel members received an e‐mail inviting them to participate. A further 20 panel members recruited for a recently cancelled FG organised by Nivel were also invited to participate in this study.

Of the individuals who were willing and able to participate, seven participants were selected per FG based on gender, age category, education level and place of residence. The recruitment aimed for diversity within the groups. Age stratification was used, resulting in FGs for each of the following age categories: 18–45, 46–64 and 65 years and older. This categorisation was made because age was often mentioned in Dutch public debate about SARS‐CoV‐2 morbidity and mortality. The categorisation also minimised discussions becoming too focused on generational differences.

### 2.3 FG Structure and Context

An FG guide (Annex 1) was developed by L.S.K.K. and E.B. using an explorative approach. Open questions were asked to capture insights into the knowledge of and concerns about the COVID‐19 pandemic and the non‐pharmaceutical interventions in place at the time. The questions were designed to gain citizen views of the actors involved in the COVID‐19 pandemic response. The FG guide was discussed and finalised with A.C.G.V., M.C.D.V., A.E.M.B., M.P.G.K. and A.T.

The structure of the three FGs was identical and consisted of three parts, as shown in Table 1. The first part consisted of an introduction round, within which participants and moderators introduced themselves. The moderators then introduced the goals and structure for the session. Verbal consent was obtained to record the FGs.

**Table 1:** The components of the focus group guide.

Part	Aim	Aspects
1	Introduction	Introduction of participants and moderators, as well as focus group goals and structure
2	Warming up	Questions about participants' knowledge and concerns about the COVID‐19 pandemic
3	Identifying the actors involved in the COVID‐19 pandemic response	1. Filling out a table naming actors they believe are involved 2. Watching a short video introducing some actors involved in COVID‐19 pandemic response 3. Studying a mind map of potential actors involved in COVID‐19 pandemic response 4. Discussion of the actors involved in the Dutch COVID‐19 pandemic response

In the second part, warming‐up questions were asked about the participants' knowledge of, and concerns about, the COVID‐19 pandemic. These questions were asked to encourage the participants to think about the pandemic generally, before discussing the actors in depth. The answers to these questions also helped put later responses into context. In the third part, the actors involved in the COVID‐19 pandemic response were discussed. To start, participants were asked to fill in a table with actors they thought were involved in the COVID‐19 pandemic response. Actors could be organisations or individuals at subnational, national or international levels. Then, participants watched a short video made by the Dutch MoHWS. The video introduced some of the many actors involved in the COVID‐19 pandemic response. Participants were also presented with a mind map designed by the Netherlands Institute for Public Safety (NIPS). The mind map showed the potential actors involved in an outbreak response. The materials used during the FGs can be found in Annexes 2 and 3.

Based on the completed tables and the information presented, the actors involved in the COVID‐19 response were extensively discussed. Lastly, participants were asked to reflect on their role, as citizens, during the response.

All sessions were conducted online, using the GoToMeeting software. They were conducted in Dutch and lasted approximately two hours each. Two researchers (L.S.K.K. with E.B. or S.K.) moderated the sessions.

### 2.4 Data Analysis

The recorded sessions were transcribed verbatim. An inductive thematic analysis was performed using the MAXQDA 2020 software. Using the process of open and axial coding [18], two independent researchers coded meaningful fragments of one of the FG's transcripts (L.S.K.K. and L.C.v.E.), and the coded texts were systematically compared by the researchers (L.S.K.K. and L.C.v.E.). The differences and similarities were analysed (L.S.K.K.) and discussed (L.S.K.K. and L.C.v.E.) and improved (L.S.K.K. and L.C.v.E.). This was repeated for the other two FGs. Using the process of selective coding [18], emerging themes and subthemes were identified by one researcher (L.S.K.K.) and checked by another (L.C.v.E.).

The results were reported following the Consolidated Criteria for Reporting Qualitative Research (COREQ) checklist [19].

### 2.5 Ethics Approval and Consent to Participate

The study protocol (LCI‐444) was reviewed by the Clinical Expertise Centre of the NIPHE. Based on this review, they determined that the research plan does not fall under the scope of the Dutch law on medical research involving humans (WMO).

All participants provided audio‐recorded oral consent for their participation in the FGs, which was documented in the transcripts. All participants were informed of the intention to publish the anonymised results.

## 3 Results

### 3.1 Demographics

Three FGs with Dutch citizens, each lasting approximately two hours, were held on 4, 5 and 9 June 2020. A total of 16 participants took part in the FGs. Due to several last‐minute cancellations, each session had five or six participants. The stratification and characteristics of the FG participants are presented in Table 2.

**Table 2:** Participant demographics.

	FG1: 18–45 years old	FG2: 46–64 years old	FG3: 65 years and older	All (%)
Participants (*n*)	5	5	6	16
Age range (years)	27–37	48–64	65–78	27–78
Gender				
Male	2	3	5	10 (62.5%)
Female	3	2	1	6 (37.5%)
Education^a^				
Early childhood education, primary education or lower secondary education	0	0	0	0 (0%)
Upper secondary education or postsecondary/non‐tertiary education	1	0	1	2 (12.5%)
Short cycle tertiary education, bachelor (or equivalent), master (or equivalent) or doctoral (or equivalent)	4	5	4	13 (81.3%)
Unknown	0	0	1	1 (6.3%)
Region of residency				
North	1	0	1	2 (12.5%)
East	1	2	1	4 (25%)
South	0	2	1	3 (18.8%)
West	3	1	3	7 (43.8%)
Place of residence				
Very strongly urban	1	1	2	4 (25%)
Strongly urban	2	1	0	3 (18.8%)
Moderately urban	2	0	0	2 (12.5%)
Slightly urban	0	3	2	5 (31.2%)
Not urban	0	0	2	2 (12.5%)

^a^ Education is classified using UNESCO's 2011 International Classification of Education (ISCED).

### 3.2 Major Themes

The analysis of the FGs resulted in the identification of three major themes across the three FGs. The identified themes are (1) the citizen's incomplete awareness of the scope of actors involved in the COVID‐19 pandemic response, (2) the need for information on actors involved in the COVID‐19 pandemic response and (3) the citizen's limited role in the COVID‐19 pandemic response. Supportive quotes from the three FGs are provided in tables for each theme.

#### 3.2.1 The Citizen's Incomplete Awareness of the Scope of Actors Involved in the COVID‐19 Pandemic Response

All participants could identify some actors they thought were involved in the COVID‐19 pandemic response. Yet, none of the participants were able to provide a comprehensive list of actors, as presented in the video and mind map.

The majority of the 16 participants could identify the following four actors:

- Fourteen participants named the NIPHE (or individuals representing the institute)
- Thirteen named the government (or its ministries or individuals representing the national government)
- Twelve named Municipal Health Services (MHSS)
- Eleven named the OMT

Other actors who were named by less than half of the participants included specific subnational governance structures (or representatives such as mayors), as well as universities, laboratories, high‐profile virologists and intensive care specialists, other healthcare providing individuals or institutions, those responsible for coordinating resources, and the police.

Non‐governmental and non‐healthcare organisations were barely mentioned, and there was no mention of the PAC.

A comparison of the age categories shows that participants from the 46–64 years age category collectively named the most number of actors in their tables. They named an average of 10.4 actors per person. The participants from the 18–45 years age category named the least number of actors, with an average of 5.6 actors named per person. On the one hand, it appears that some individuals from the 46–64 years age category attempted to adopt to a systematic approach to naming the actors, as shown in Table 3. On the other hand, some participants from the 18–45 years age category specifically acknowledged that they were likely to think of actors they believed had received a lot of media attention.

**Table 3:** Supportive quotes showing different approaches to naming actors.

	Quotation
18–45 years old	*I have actually only written down people you often see on the news, talk shows […]*
46–64 years old	*[…] You can look for the complicated aspect and consider everything. Or you can look for something you can use, that can serve as an instrument[…] Only I was not completely satisfied with the order when I started writing.*
65 years and older	N/A

Furthermore, when discussing the actors named by the participants and those presented in the video and mind map, participants from all three age categories noted that it was unclear to them what the specific roles and tasks of these actors' were within the COVID‐19 management structure. Supportive quotes are shown in Table 4.

**Table 4:** Supportive quotes showing the lack of clarity of roles and tasks of actors.

	Quotation
18–45 years old	*[…] I realise that I do not really know what everyone does exactly. Look, I have the NIPHE on my list, but I cannot easily state what their activities are.*
46–64 years old	*[…] I think everything can think of a role for every actor, but i have not been able to identify a structure […]*
65 years and older	*When I see this diagram […] there are so many organisations and people involved. Are they involved? Are they not simultaneously doing the same thing? There are so many duplications. Who decides who does what? And aren't they not looking for the same?*

Most participants stated that there was an information overload concerning the COVID‐19 pandemic. However, there seemed to be little easily accessible information available on the actors involved in the COVID‐19 response. Yet, the need to have information on the constellation of actors was expressed in the FGs.

#### 3.2.2 The Need for Information on Actors Involved in the COVID‐19 Pandemic Response

The second theme focused on the reasons why participants would like to have information on the actors involved in the COVID‐19 pandemic response. Some participants stated two reasons why they would have appreciated more information on which actors were involved.

First, some participants stated that it could help them evaluate and form an opinion on the COVID‐19 pandemic response. They expressed a desire to be better informed of the actors involved in the pandemic response, especially those in charge. It would help them understand the role and relevance of the vast number of actors involved. It was stated that this could also help to have a better appreciation of factors that played a role in policy‐making. Supportive quotes for these opinions are shown in Table 5.

**Table 5:** Supportive quotes for participants' need for information on actors to evaluate the COVID‐19 response.

	Quotation
18–45 years old	*[…] I think it would be good to know which hospitals are involved, but also the business aspect. Because we have very much focused on health, which I think is important. But what about nursing homes, for example? I think there are other interests there than in hospitals […] That kind of information is lacking, especially the transparency.*
46–64 years old	*It was not the intention at first and then the school closed under societal pressure. The NIPHE's researchers, the Outbreak Management Team they all said or believed that it was not necessary. Even the Cabinet said, ‘We do not think it is necessary’. But yes, the people really wanted it and it happened. Then when it was being considered how to relax the interventions, the first intervention relaxed was opening the schools. And when the schools were opening, people were emphasising the 1.5 m rule. Whereas it was never the intention to close the schools. I do not follow why it happened like this. I do not understand it at all. So, I would like to discuss that with someone at some point; to understand how such decisions are made.*
65 years and older	*This diagram shows how complex managing the crisis was and is […] When you see this, it becomes complicated, as there are so many people, actors and groups involved, who have their individual interests. I think balancing those can be challenging.*

Second, some participants from the 18–45 years age category particularly stated that it would increase their acceptance of non‐pharmaceutical interventions implemented. This is illustrated in the following quote:

I think the list, like the one on the screen[…] I think understanding, or dissatisfaction will be reduced for a lot of people if you know how many people are involved behind the screens. Because if I indeed look at the interventions, then they feel imposed, and many people are dissatisfied. If you can show them this overview and you can see what happens behind the screens, I can imagine that people maybe think ‘maybe many parties have put a lot of thought into it’ and it will increase satisfaction.

(18–45 years old)

#### 3.2.3 The Citizen's Limited Role in the COVID‐19 Pandemic Response

The third theme referred to the fact that participants generally limited their role during PHEPR to curbing the spread of the virus. They did this by adhering to the non‐pharmaceutical interventions introduced. As shown in the earlier quote, some participants believed public pressure could result in reversing the implementation of certain non‐pharmaceutical interventions. Yet, when specifically asked which actors were involved in the COVID‐19 pandemic response, only three of the 16 participants spontaneously mentioned the citizen (or a synonym). Even when encouraged to think about this specific aspect, most participants primarily noted that every member of society has a responsibility in the pandemic response. Their responsibility is to avoid being infected and to avoid infecting others, as shown in Table 6.

**Table 6:** Supportive quotes showing participants predominantly adhering to non‐pharmaceutical interventions.

	Quotation
18–45 years old	*I comply with the NIPHE interventions. I do what I can […] and keep myself as healthy as possible. I assume that the people in my surroundings and I will stay healthy.*
46–64 years old	*We have done a lot of social distancing, which was quite difficult because our grandchildren will turn one this week. That is an age when you would really like to have them close to you, but you just do not do it. Imagine if you transmit something.*
65 years and older	*I think we actually have to do it together by sticking to the rules until there is a vaccine. It is the only option for now.*

Participants had varying opinions on the extent to which they wanted to engage with decision‐makers during the COVID‐19 pandemic response. Some participants, especially in the 18–45 and 65 years and older age categories, stated that they did not have the desire to share their opinions with decision‐makers. They preferred to limit the expression of their opinions within their private circles and suggested that the experts with the necessary knowledge were already involved, as shown in Table 7.

**Table 7:** Supportive quotes showing some participants do not wish to publicly express their opinion.

	Quotation
18–45 years old	*I do not think I felt the need to share my opinion, especially in the beginning. You assume that specialists make good decisions for you. Yes, I think that it is like football. When there is a football match, we have 17 million coaches. Everyone has an opinion, but you do not always have to announce it publicly.*
46–64 years old	N/A
65 years and older	*If you have a problem or if you express an opinion in the Netherlands, there are approximately sixteen million other opinions. It is like football; we have sixteen million football coaches in the Netherlands, so it is useless. Look, if you want to talk about it, it is logical, it is normal, you practically only hear about it. And when you talk to someone on the phone, you also talk about it […] But I would say, start within the family, there you can express what is bothering you. But there has not been a day when I have felt the need to seek a platform or to exchange views on how I think the coronavirus should be managed.*

Yet, other participants wished to state and share their questions or opinions with decision‐makers, as shown in Table 8. They believed that engaging with decision‐makers would help them understand the decision‐making process. It would also encourage decision‐makers to consider the real‐life implications of policies implemented. However, those participants also stated that they had not been able to identify platforms or channels where they could express their opinions at a political level.

**Table 8:** Supportive quotes showing some participants suggest a need for a platform on which different segments of society can engage with decision‐makers.

	Quotation
18–45 years old	*Indeed, if you speak to citizens then you will be informed of practical problems that one may face in practice […]*
46–64 years old	*[…] What I also think is that if you have certain ideas or questions, it is imposed from above. And you also see it in the briefings; there are people often sitting behind their desks and they say certain things. But you do not have anyone from the field. Yes, in nursing you ask the professional association certain questions. And they gather opinions and experiences of those in the field. But in our organisation you see that there is also a similar crisis team who are taking the decisions.*
65 years and older	*But which platforms or where could I go to ventilate that opinion in a way it could have an impact on what happens, or which interventions are suggested. But where do I go with those views?*

## 4 Discussion

This study aimed to offer preliminary insights into the Dutch citizen's understanding and perception of the actors involved in the Dutch COVID‐19 pandemic response early in the pandemic. Despite some differences in the points emphasized in the results across the three age categories, the overarching themes were identified in all three FGs. The results suggest that the study's participants were not fully aware of the scope of actors involved in the COVID‐19 pandemic response. Only four actors were named by the majority of the participants: the NIPHE, the Government, MHSS, and the OMT. Some citizens would have appreciated receiving more information on the constellation of actors involved in the COVID‐19 pandemic response, as well as their corresponding roles and tasks. This would have helped them understand how decisions were made and increase the acceptance of those decisions. Although the COVID‐19 pandemic was a PHE of unprecedented magnitude, resulting in global continuous media attention for months, the study participants still expressed a need for transparency regarding the COVID‐19 pandemic response.

Transparency in policy‐making and within organisations has received attention in international relations, non‐profit, public policy and administration literature. Ball's [20] examination of the literature identified three definitions for transparency. The first definition is that of transparency as a public value; transparency would be accepted by society as an attempt to counter corruption. The second definition relates to the extent to which governments and non‐profit organisations are open in their decision‐making. In the third definition, transparency is complex and a tool of good governance. Transparency entails the understanding of the actors' making decisions and the nature of the decisions being made, as well as how to use the information available. Furthermore, transparency is correlated with accountability, effectiveness and efficiency. This third definition is reflected most accurately within this study, as participants expressed a desire to understand who makes the decisions, the reasoning behind the constellation of actors involved, how decisions are being made and which information is available for the decision‐making actors. The study participants suggest that this would help their ability to understand the decisions made.

One may wonder whether the study participants' wish for transparency reflects a low level of trust in the government. Although predominantly open questions were asked during the FGs, trust was not an issue that was mentioned by the participants. It was also not identified as an emergent theme during analysis. A survey study by de Vries et al. [21] showed that Dutch respondents generally had a relatively high level of trust in the Dutch authorities. This was in terms of the information provided and the interventions implemented to control the spread of the SARS‐CoV‐2 virus. The study was conducted amongst Dutch citizens during the first 3 months of the pandemic in 2020. Another study by Rieger and Wang [22], analysing a large international online survey by Fetzer et al. [23] conducted in March and April 2020, showed comparable results. The Dutch survey respondents did not have low scores in their trust in the government (score of 3.89, with 1 = *strongly distrust* to 5 = *strongly trust*) or in their perception of their country's reaction to the pandemic (score of 2.94, with 1 = *very poorly* to 4 = *very well*). These results suggest that this study participants' need for transparency cannot be simply explained by a lack of trust in the government.

Besides needing transparency, most study participants did not spontaneously consider themselves actors in the COVID‐19 pandemic response. Yet, during in‐depth discussions on this topic, participants predominantly mentioned their responsibility to avoid getting infected and infecting others. This finding is in line with Kuiper et al.'s [12] findings from an online survey conducted in April 2020 in the Netherlands. The study showed high levels of reported compliance.

Participants did not suggest a role for themselves as citizens in the COVID‐19 response *decision‐making*. Yet, some did believe that if decision‐makers listened to different segments of society, it could increase the feasibility of decisions made. However, good governance literature generally encourages deeper citizen involvement in public policy‐making processes [24, 25] than what was discussed by this study's participants. The literature suggests that citizens should be engaged and not simply considered service users [26]. This suggestion is in line with WHO's whole‐of‐society approach, as described in the WHO's 2017 Strategic Framework for emergency preparedness [15]. The document stresses the need for multisectoral collaboration and highlights the role of community members. It states that the community should be represented in all emergency preparedness activities. This echoes the WHO 2009 Guideline for pandemic preparedness and response in the non‐health sectors [14]. Civil society is considered one of the three central sectors in the guideline. The other two sectors are the government and business sectors. Our research on identifying the sectors described in European pre‐COVID‐19 pandemic PHEPR literature [27] shows that civil society was one of four commonly mentioned sectors. Yet, it was mentioned less often than governmental institutions and the human health industry. Furthermore, a consensus study conducted in 2022 showed that European PHEPR experts believed the sector ‘civil society’ should be included in PHEPR decision‐making [28].

Despite the assumption that citizens should be included in decision‐making during PHEs, it remained unclear how this should take place and how citizens feel about it. The study's findings showed that not all participants wished to express their opinions or engage with decision‐makers. This is in line with the findings of an FG study also conducted in June 2020 by Kemper et al. [29]. The study showed that most Dutch participants stated that their role in outbreak management is passive, and they expected to receive information. This was confirmed by a survey conducted amongst a representative sample of the Dutch public approximately 5 months later [30]. It showed that only 25% of respondents expressed a desire to engage in decision‐making.

### 4.1 Strengths and Limitations

This study contributes to the sparse literature on the citizen's perspective on PHEPR. It offers preliminary insights to be considered when aiming for citizen participation in PHEPR when applying the whole‐of‐society approach.

Most of this study's authors have been involved in the COVID‐19 pandemic response. This allowed for the possibility of discussions of the study outcomes within policy‐relevant settings. However, the authors were also mindful of the potential influence their affiliations could have on their roles as researchers and moderators. The participants were informed of the moderators' affiliated institutions, but it is unlikely that the influence of these affiliations was big. The participants did not seem to give socially desirable answers. On the contrary, they were at times critical of the moderators' affiliated institutions.

The study was designed shortly after the start of the pandemic. There was a lot unknown about the SARS‐CoV‐2 virus and it was quickly spreading. Planning and conducting this study in a short amount of time during an unprecedented lockdown had its logistical limitations. Limitations included switching from live to online FGs for the first time. The timing of the study allowed for the collection of valid results with minimal recall bias. However, it also meant limited time for participant recruitment.

We acknowledge that the use of an existing panel may favour the inclusion of participants interested in sharing their opinions about healthcare. However, it must also be noted that individuals are invited to join the DHCCP and cannot simply sign up for it. Despite efforts to select the participants to ensure diversity, non‐immigrants and people with higher education were over‐represented in the study. Hence, future research on the topic should aim to employ alternative recruitment methods to achieve more diversity. This would help to evaluate the applicability of this study's results in other settings.

### 4.2 Future Research

To have a deeper understanding of the citizen's perspective on PHEPR, more research must be conducted while focussing on PHEs of other origins. Research should also be conducted in a larger geographic context. Both situations may provide insights into PHE characteristics and contextual factors that may influence the citizen's perspective.

Furthermore, conducting research *after* the COVID‐19 pandemic may be informative. It can help evaluate whether the Dutch citizen's understanding of the actors in the COVID‐19 response has been influenced by (i) the information that is now available and (ii) the experiences lived during and since the COVID‐19 pandemic.

## 5 Conclusion

This study suggests that the Dutch citizens participating in this study's FGs did not have a full understanding of the scope of (potential) actors involved in the Dutch COVID‐19 pandemic response. The insight captured can inform debate about public involvement and engagement in preparedness and response. The study shows a need for more information on the pandemic response. This will help participants better evaluate the actors involved and understand the non‐pharmaceutical interventions implemented.

## Author Contributions

**L.S. Kengne Kamga:** conceptualisation, methodology, writing–review and editing, writing–original draft, project administration, formal analysis, data curation, investigation. **A.C.G. Voordouw:** conceptualisation, supervision, writing–review and editing, methodology, funding acquisition. **M.C. De Vries:** conceptualisation, writing–review and editing, supervision, methodology, funding acquisition. **E. Belfroid:** conceptualisation, methodology, investigation, supervision, writing–review and editing. **A.E.M. Brabers:** methodology, writing–review and editing, investigation. **J.D. De Jong:** methodology, writing–review and editing. **L.C. van Eck:** writing–review and editing, formal analysis. **M.P.G. Koopmans:** conceptualisation, writing–review and editing, supervision, methodology, funding acquisition. **A. Timen:** conceptualisation, methodology, funding acquisition, writing–review and editing, supervision.

## Ethics Statement

The study protocol was approved by the Centre for Clinical Expertise at the National Institute for Public Health and the Environment, with study protocol number LCI‐444.

## Consent

All study participants provided oral consent.

## Conflicts of Interest

The authors declare no conflicts of interest.

## Supporting information

Supporting information.

Supporting information.

Supporting information.

## Data Availability

Due to the sensitive nature of the study, the data set analysed during the study is not publicly available. It is available from the corresponding author upon reasonable request.
