# Supplementary material for: The Dutch Citizen's Understanding and Perception of the Actors Involved in the Netherlands' COVID‐19 Pandemic Response: A Focus Group Study During the First Pandemic Wave
Source: Health Expect. 2024 Sep 6;27(5):e14170. doi: 10.1111/hex.14170 (PMC11377844; doi:10.1111/hex.14170)
Supplement: Supplementary file 1 — Supporting information. [file HEX-27-e14170-s001.doc]

DRAAIBOEK FOCUSGROEP DISCUSSIE

# The citizen’s perception of the Netherland’s national COVID-19 preparedness and response

Ten behoeve van de focusgroep discussie met burgers in juni 2020

Contact: Sandra Kamga (sandra.kengne.kamga.mobou@rivm.nl)

18-05-2020

**Afkortingen**

**FGD** Focusgroep discussie

**LCI** Landelijke Coördinatie Infectieziektebestrijding

**RIVM** Rijksinstituut voor Volksgezondheid en Milieu

# Inleiding

De focusgroep discussies (FGD) zullen gehouden worden ten behoeve van het onderzoek dat wordt uitgevoerd door promovendus Sandra Kamga, in opdracht van het EU Joint Action SHARP. Tijdens dit onderzoek zullen de onderzoekers de meningen, ideeën en ervaringen van de burger over de COVID-19 uitbraakbestrijding inventariseren en uitvragen.

Achtergrondinformatie Online Bijeenkomst

Een FDG is een geschikte methode om de meningen en ervaringen van bepaalde groepen mensen over een specifiek onderwerp te verzamelen. Tijdens een FGD is er een constructivistische benadering. Er is geen absolute waarheid en de realiteit die beschreven wordt tijdens de dataverzameling is de realiteit zoals dat de individuen en de groep ervaren. De informele context van een FGD zou de participanten moeten aanmoedigen om makkelijker hun meningen en ervaringen te delen. Het is net het doel om consensus te bereiken maar ideeën uit te wisselen. De voordelen van focusgroepen zijn dat de onderzoeker in één keer informatie kan verzamelen over meerdere individuen tegelijk, en dat er ook informatie over drie niveaus, namelijk de individu, de groep en de interactie onderling, verzameld kan worden.

Doel van de focusgroepsdiscussie

Tijdens deze focusgroepen zal data verzameld worden om de volgende 2 onderzoeksdoelen te beantwoorden:

1. Explore to what extent Dutch citizens have an understanding of the Dutch COVID-19 crisis management structure
2. What are the Dutch citizens’ perceptions of the Dutch COVID-19 Dutch crisis management structure is

Focusgroep discussie

Er zullen in totaal drie focusgroepen van 5 à 7 man plaatsvinden, verspreid over 3 dagen. Een focusgroep zal participanten onder de 45 jaar includeren, een andere participanten tussen de 46 en 64 jaar en de laatste participanten van jaar en ouder.

Een moderator en een assistent leiden de FDG. Zij zorgen dat de kaders van het gesprek duidelijk zijn, zij faciliteren waar nodig het gesprek en zij observeren en noteren de opmerkingen en gedrag van de participanten. Het is niet bedoeling dat het, zoals bij een interview, een vraag-antwoord sessie wordt maar dat juist de participanten veel aan het woord zijn en onderling op elkaar reageren (zie bijlage 2)

**Focusgroep 1:**

**Moderator**: Evelien Belfroid

**Assistent:** Sandra Kamga

**Focusgroep 2:**

**Moderator**: Sandra Kamga

**Assistent:** Evelien Belfroid

**Focusgroep 3:**

**Moderator**: Sandra Kamga

**Assistent:** Sophie Kemper

**Respondenten**

Participanten

Er wordt gepoogd om een diverse groep van 21 burgers te verzamelen die deel zullen nemen aan de een van de bijeenkomsten in juni 2020. Er wordt gekeken naar geslecht, leeftijd en opleidingsniveau. Dit is van belang om zo veel mogelijke meningen (en de bijbehorende onderbouwing) te horen die de Nederlandse burgers hebben.

Benadering participanten:

- 1000 willekeurige leden van de Nivel panel (een consumentenpanel 12.000 Nederlanders van boven de 18 die door de selectiemethode representatief is voor de Nederlandse volwassenen wat betreft geslacht, leeftijd en opleidingsniveau) zijn door Nivel schriftelijk uitgenodigd om mee te doen met de Burgerplatform met een de focusgroep die die bij hen leeftijd aansluit.
- Nivel heeft reacties hierop ontvangen en heeft daarvan 21 burgers geselecteerd om daadwerkelijk mee te doen.

Faciliteiten

- De drie FGD zullen digitaal plaatvinden GoToMeeting

# Draaiboek online bijeenkomst

Hieronder wordt stap-voor-stap de inhoud van de focusgroep discussie beschreven.

WELKOMSWOORD EN INTRODUCTIE (10 minuten)

Moderator: Goedemorgen allemaal, bedankt voor jullie aanwezigheid tijdens deze bijeenkomst. Als eerste wil ik vragen of ik jullie met je mag aanspreken?

Ik zal mij eerst voorstellen: Mijn naam is Sandra. Ik kom uit Den Haag en woon in Utrecht. Ik ben onderzoeker bij het RIVM. Ik doe onderzoek naar volksgezondheid crises. Daarom heb jullie vandaag uitgenodigd omdat ik benieuwd ben naar in hoeverre het duidelijk is wie wat doet gedurende de bestrijding van Corona en wat jullie daarvan vinden.

Ik ben hier vandaag met mijn collega Evelien/ Sophie van het RIVM en Laurens/Anne van Nivel.

[Assistent stelt zich voor].

[Nivel stelt zich voor].

Wij zijn natuurlijk erg blij dat jullie hier zijn en wij zijn benieuwd naar wie jullie zijn. [Ik wil graag iedereen vragen om zijn camera aan te zetten]. Ik ga mijn lijstje af van aanwezigen en als ik je naam roep kan jij jezelf voorstellen met je naam, woonplaats, beroep en hobby.

[Deelnemers stellen zich voor]

Leuk om kennis met jullie te maken! Zoals ik eerder aangaf, ben ik benieuwd naar jullie mening over de Nederlandse aanpak gedurende de Corona crisis. Tijdens deze groepsdiscussie willen wij graag in kaart brengen wat jullie ideeën en meningen zijn over hoe de Corona-virus wordt bestreden in Nederland. Wij horen graag alle ideeën, meningen en persoonlijke ervaringen van jullie allemaal. Er zijn geen goede en foute antwoorden of meningen. Jullie hoeven niet met elkaar eens te zijn. Als jullie zelf vragen hebben kunnen jullie ze in de groep stellen en kan iedereen daarop reageren.

Ik ben de discussieleider en ben er voornamelijk om de discussie te sturen en tempo in de discussie te houden. Jullie praten, ik stel af en toe een vraag en vat wat jullie zeggen samen.

De discussie die wij voeren is onderdeel van mijn onderzoek en de uitkomsten wil ik publiceren. Als je het rapport per mail zou willen ontvangen kan je nu je hand opsteken. Om te zorgen dat wij niets missen en de hele discussie kunnen gebruiken voor dit onderzoek gaan wij de discussie opnemen. Gedurende de uitwerking worden alle namen verwijderd zodat uitspraken niet herleidbaar zullen zijn. Jullie hebben als het goed is een e-mail hierover ontvangen. Zijn er mensen die bezwaar hebben tegen deze opname? Zo ja, kunt u het gesprek verlaten. Zo niet, ga ik nu de opname starten.

Die zet ik vanaf dit moment aan!

**[Opname]**

Ik ga nu namen oproepen zodat het ook vastgelegd is dat jullie bezwaar hebben tegen het opname van deze sessie en voor het gebruiken van de informatie voortkomend uit dit gesprek voor publicaties. Als je naam hoort is een ‘geen bezwaar’ voldoende.

Deze groepsdiscussie zal ongeveer anderhalf uur lang zijn, met een pauze tussendoor.

Informatie over Bol.com cadeaubon sturen we na afloop vanuit het Nivel op.

Zijn er nog vragen? Alles duidelijk?

Een paar instructies om dit digitaal gesprek zo soepel mogelijk te laten verlopen:

- Camera’s aan
- Microfoon uit indien je niet praat
- Hand opsteken als je iets wilt zeggen. In de chat iets sturen mag ook.

VRAAG 1 (10 MINUTEN)

Doel: Hun meningen te horen over de informatievoorziening w.b.t. de COVID-19 en de bestrijding en om hun antwoorden te kunnen contextualiseren.

**De moderator zegt:**

*Horen of lezen jullie informatie over de Corona-uitbraak (en zo ja, wat vinden jullie ervan)?*

**Subvragen (graag alle subvragen doorlopen!):**

- *Gaan jullie zelf actief op zoek naar informatie?*
- *Wat voor informatie horen en/of lezen jullie?*
- *Wat vinden jullie van de informatie die jullie horen en/of lezen?*
- *Is de informatie duidelijk?*
- *Zijn er onderwerpen of onderdelen van de corona-uitbraak en bestrijding waar jullie meer informatie over zouden willen ontvangen (en zo ja, welke)?*

VRAAG 2 (10 MINUTEN)

Doel: Een beeld te schetsten over of de Corona-uitbraak bij burgers speelt en wat hun belangrijkste zorgen over de Corona-virus zijn, en om ook hun antwoorden te kunnen contextualiseren.

**De moderator zegt:**

*Maken jullie jullie zorgen over het Coronavirus?*

**Subvragen:**

- **Bij elk antwoord vragen:** *Waarom maak je je wel of geen zorgen?*
- ***Indien zij alleen ja zeggen zonder verder uitleg:***
  - *Om wie maak je je zorgen?*
  - *Om wat maak je je zorgen?*

TABEL: PERSONEN EN ORGANISATEIS BETROKKEN BIJ DE COVID-19 uitbraak (10 minuten)

De moderator zal de participanten vragen om een tabel te maken over welke personen en organisaties zij denken betrokken zijn bij de bestrijding van de COVID-19 uitbraak.

De assistent notuleert interessante opmerkingen en vragen die gesteld worden en houdt de tijd in de gaten. Er is in totaal 20 minuten beschikbaar voor dit onderdeel.

1. **Om uit te leggen hoe zij hun tabel moeten invullen zegt de moderator:**

*Wij weten natuurlijk dat er verschillende mensen, groepen en organisaties bezig zijn met het bestrijden van het Coronavirus. Hier gaan wij het even over hebben doormiddels van een activiteit.*

*Ik ga mijn scherm met jullie delen om te laten zien hoe wij dit gaan doen. Wat hebben jullie nodig? Jullie hebben per mail een tabel ontvangen zoals ik hier op mijn scherm heb. Indien je het niet kan vinden kan je ook op een wit paper of in een Word iets soortgelijks tekenen, Die mogen jullie gebruiken. In de linker kolom mogen jullie alle personen, groepen en organisatie opschrijven die volgens jullie dat er betrokken zijn bij het bestrijden van deze uitbraak en in de rechter kolom mogen jullie schrijven wat jullie denken dat zij doen. We geven jullie 10 minuten de tijd om iedereen die jullie denken dat betrokken zijn op het papier te schrijven. Jullie mogen alle personen en organisaties opschrijven ook al weten jullie niet precies wie zij zijn en wat zij doen. En jullie kunnen ook een activiteit opschrijven ook al weten jullie niet precies wie dat doet. Op mijn gedeelde scherm zien jullie een kort voorbeeld. Het is ook prima als je geen of weinig personen of organisaties weet. We willen gewoon heel graag weten wie jullie denken dat er betrokken zijn.*

***[10 min werken aan mindmap]***

VRAAG 3 (10 MINUTEN)

Doel: De diepte in te gaan over hun kennis over de samenwerkingsstructuur in Nederland en wat zij zelf opmerking.

**De moderator zegt:**

*Dank voor het invullen van de tabel. Wij komen hier zo op terug. Nu wil ik vragen in hoeverre is het voor jullie duidelijk wie wat doet en wanneer in de Coronabestrijding?*

**Subvragen (graag alle subvragen doorlopen!):**

- ***Is het voor jullie duidelijk welke personen, groepen en organisaties betrokken zijn bij de bestrijding van de Corona-uitbraak?***
  - *Zo ja, hoe is dit duidelijk geworden voor jullie?*
  - *Zo nee, wat zou nodig zijn om het duidelijker te maken?*
- ***Weet je welke personen, groepen en organisaties samen werken?***
  - *Zo ja, hoe is dit duidelijk geworden voor jullie?*
  - *Zo nee, wat zou nodig zijn om het duidelijker te maken?*
- ***Weet je welke organisatie wat doet?***
  - *Zo ja, hoe is dit duidelijk geworden voor jullie?*
  - *Zo nee, wat zou nodig zijn om het duidelijker te maken?*
- *Concluderend: hebben jullie het idee dat jullie een volledig overzicht van wie wat doet.*

PRESENTATIE (10 MINUTEN)

*Nu wil ik jullie een kort filmpje laten zien.*

Tijdens deze presentatie zal Sandra een kort filmpje van het RIVM tonen, gevolgd door een mindmap van VWS laten zien (zie PowerPointpresentatie). Het doel van deze presentatie is om aan de geven dat er veel partijen betrokken zijn en dat het een complexe situatie is.

1. ***Na het filmpje zegt Sandra:***

*In het filmpje hebben jullie kunnen zien dat er meerdere mensen en organisatie betrokken zijn bij de bestrijding van de Corona-uitbraak. Er zijn veel taken die opgepakt moeten worden om te zorgen dat de bestrijding zo goed mogelijk verloopt en dat het normale leven door kan blijven gaan. Dit vereist de kennis en kunde van verschillende mensen en organisaties. Dit plaatje die op het scherm staat.*

**Tonen mindmap van het Instituut Fysieke Veiligheid**

*In dit plaatje zien jullie dat er meerdere personen en organisatie betrokken zijn bij de bestrijding van uitbraken. Er zijn natuurlijk altijd een aantal mensen en organisatie die meer op de voorgrond staan en een zwaarder takenpakket hebben. Wij zijn voornamelijk benieuwd wie jullie denken dat dat zijn en wat jullie mening is over hoe de bestrijding verloopt.*

VRAAG 4 (10 MINUTEN)

Doel: De diepte in te gaan over hun kennis over de samenwerkingsstructuur in Nederland en wat zij zelf opmerking.

**De moderator zegt:**

*Jullie hebben jullie tabel ingevuld en jullie zien een andere mindmap die laat zien wie bij de Corona-uitbraak bestrijding betrokken zijn. Wat valt op als jullie deze mindmap vergelijkt met jullie tabellen?*

*Mindmap delen op scherm*

**Subvragen:**

- ***Als er weinig reactie is:***
  - *Vinden dat er belangrijke personen, groepen of organisaties ontbreken?*
  - *Wat zijn de overeenkomsten?*
  - *Wat zijn de verschillen?*
- **KORTE SAMENVATTING door de moderator**
- **PAUZE (10 MINUTEN)**

Vraag 5 (10 MINUTEN)

Doel: De meningen horen over de algemene COVID-19 bestrijding horen en voornamelijk de uitkomsten te horen.

**De moderator zegt:**

*Hoe vinden jullie dat de bestrijding van de Corona-uitbraak in Nederland gaat? Gaat het goed of gaat het niet goed?*

**Subvragen**:

- **Bij alle antwoorden vragen:** *Waarom vind jij dat het wel/niet goed gaat?*

VRAAG 6 (10 MINUTEN)

Doel: Om een beeld te krijgen of zij inzicht hebben in de gewenste uitkomsten van de Corona-bestrijding.

**De moderator zegt:**

*Wat denken jullie dat de personen, groepen en organisaties die betrokken zijn bij Corona-bestrijding willen bereiken?*

**Subvragen**:

- - **Indien ze het weten:**
    - Wat willen zij bereiken?
    - Hoe weten jullie dit?
  - **Als het niet duidelijk is:** Wat moeten deze personen, groepen en organisaties doen om hun doelen duidelijker te maken?

VRAAG 7 (10 MINUTEN)

Doel: Om een beeld te krijgen of zij vinden dat de samenwerkingsstructuur naar hun behoeftes luistert.

**De moderator zegt:**

*Een heel groot deel van de beslissingen die gemaakt worden tijdens deze crisis hebben een groot invloed op iedereen. Hebben jullie behoefte aan om jullie meningen over de aanpak en nog te nemen beslissingen te geven aan de betrokken personen of organisaties?*

**Subvragen:**

- **Zo ja:**
  - *Op welke manier hebben jullie dit kunnen aangeven of op welke manier zouden jullie dit willen doen?*
- *Wordt er voor jullie gevoel geluisterd naar jullie wensen en behoeftes tijdens de bestrijding van de Corona-uitbraak?*
  - *Zo ja, waarom denken jullie dat?*
  - *Hoe zou dit beter kunnen?*

AFRONDING (3 MINUTEN)

De moderator vat kort samen en vraagt of dit de juiste weerspiegeling is van wat er vanmiddag besproken is of dat er nog dingen bespreken. Daarnaast wordt er nog gevraagd naar mogelijke vragen/opmerkingen/ideeën.

**Moderator:** Hierbij ronden wij onze discussie. Zijn er nog vragen, opmerkingen of ideeën?

[Vragen die relevant zijn deze studie kan de moderator en assistent beantwoorden en voor andere vragen worden ze verwezen naar het RIVM-website].

Heel erg bedankt voor jullie bijdrage. Als een kleine blijk van waardering ontvangen jullie een Bol.com cadeaubon per e-mail vanuit het Nivel. Wij wensen jullie een fijne middag/avond verder. Misschien hier nog keer vragen of ze de tabel willen opsturen als dat nog niet gelukt is.

# Bijlage 1 Taken discussieleider en notulist

Taken van de discussieleider:

- goed luisteren, doorvragen en interpreteren
- ervoor zorgen dat er niet van het onderwerp wordt afgeweken
- zorgen dat de discussie zich ontwikkeld
- zorgen dat niet 1 persoon de leiding heeft in de discussie
- zorgen dat ieders mening gehoord wordt
- zorgen dat sleutelvragen besproken worden
- ideeën die deelnemers heel belangrijk vinden benoemen en op terug komen, maar die er niet veel toe doen in de discussie worden ‘geparkeerd’. Dit betekent dat de voorzitter het genoemde punt opschrijft en zal meenemen in de analyse maar dat er tijdens de discussie geen aandacht meer aan wordt besteed. Dit om te voorkomen dat deelnemers te lang blijven praten over niet relevante punten, maar zich niet gepasseerd voelen
- discussie op de onderwerpen laten focussen zonder dat de stroom van ideeën wordt afgekapt
- stilte mag gewoon stilte blijven, afwachten, uiteindelijk gaat er wel iemand praten, tenzij de deelnemers de vraag niet snappen. Dan vraag herhalen of op andere manier stellen (geen mogelijke antwoorden geven!).
- Open vragen stellen: bijvoorbeeld:
  - Wat vind je ervan?
  - Wat vind je van het feit dat?
  - Hoe kijk je daar tegenaan?
  - Wat is je reactie hierop?

Technieken vijf seconde pauze en verdieping, om meer informatie los te krijgen:

Vijf seconde pauze = effectief na een reactie van een deelnemer. Nodigt andere deelnemers uit om te reageren en levert vaak aanvullende gezichtspunten op. Deze pauze zorgt er ook voor dat de voorzitter niet teveel aan het woord is.

Verdieping = om vage uitspraken en commentaar te verduidelijken, voorkomt vage groepsdiscussie. Bruikbare vragen: Wilt u dit wat verder uitleggen? Wilt u mij een voorbeeld geven van wat u bedoelt? Kunt u er iets meer over zeggen?

Taken van de notulist:

- in hoofdlijnen opschrijven hoe de discussie verloopt, hoe reageren respondenten op elkaar, hoe is de sfeer
- eerste en laatste woord van wie wat zegt opschrijven?
- houdt de tijd in de gaten en geeft de voorzitter een seintje als het volgende onderdeel aan de beurt is
- nadien bespreken hoe het is gegaan, feedback en advies geven
- wie zit waar noteren
- hulp bieden in geval van problemen, als gesprek niet loopt, facilitator geen ideeën heeft, etc.

NB

**Indien er vragen ontstaan:**

*Ik kan mij voorstellen dat er wat inhoudelijk vragen zijn over hoe het georganiseerd is. Gezien wij zo weer in groepen dit onderwerp gaan bespreken en wij vooral geïnteresseerd zijn in jullie ideeën en ervaring zullen wij proberen om jullie inhoudelijk vragen pas na de focusgroep te beantwoorden. Het zal wel zinvol zijn om deze vragen onderling te bespreken tijdens de groepsdiscussies!*

*Aan het eind van de dag zullen wij kijken welke vragen jullie nog over hebben. Hou wel aub rekening mee met dat wij onderzoekers zijn en niet de beleidmakers van het RIVM, maar wij zullen de antwoorden beantwoorden die wij kunnen beantwoorden en anders jullie de juiste en actuele bronnen laten zien.*

**Indien er de burgers hier ontevreden zijn, veel vragen/zorgen hebben enz.:**

- Emoties benoemen
- Zorgen erkennen
- Aangeven dat wij vanavond hier zijn om juist naar hun meningen te luisteren
- Aangeven dat het RIVM veel informatie op de website heeft staan die wij aan het eind van de sessies kunnen laten zien
- Onze rol als onderzoekers benadrukken. Wij zijn geen beleidsmakers.
