# Supplementary material for: The Dutch Citizen's Understanding and Perception of the Actors Involved in the Netherlands' COVID‐19 Pandemic Response: A Focus Group Study During the First Pandemic Wave
Source: Health Expect. 2024 Sep 6;27(5):e14170. doi: 10.1111/hex.14170 (PMC11377844; doi:10.1111/hex.14170)
Supplement: Supplementary file 2 — Supporting information. [file HEX-27-e14170-s002.docx]

Tabel: Personen en organisatie betrokken bij de COVID-19 uitbraak

| Namen van mensen of organisatie betrokken bij de COVID-19 uitbraak | Activiteiten van persoon of organisatie betrokken bij de COVID-19 uitbraak |
| --- | --- |
|  |  |
|  |  |
|  |  |
|  |  |
