# Supplementary figures and images for: The Dutch Citizen's Understanding and Perception of the Actors Involved in the Netherlands' COVID‐19 Pandemic Response: A Focus Group Study During the First Pandemic Wave
Source: Health Expect. 2024 Sep 6;27(5):e14170. doi: 10.1111/hex.14170 (PMC11377844; doi:10.1111/hex.14170)

## Slide 1
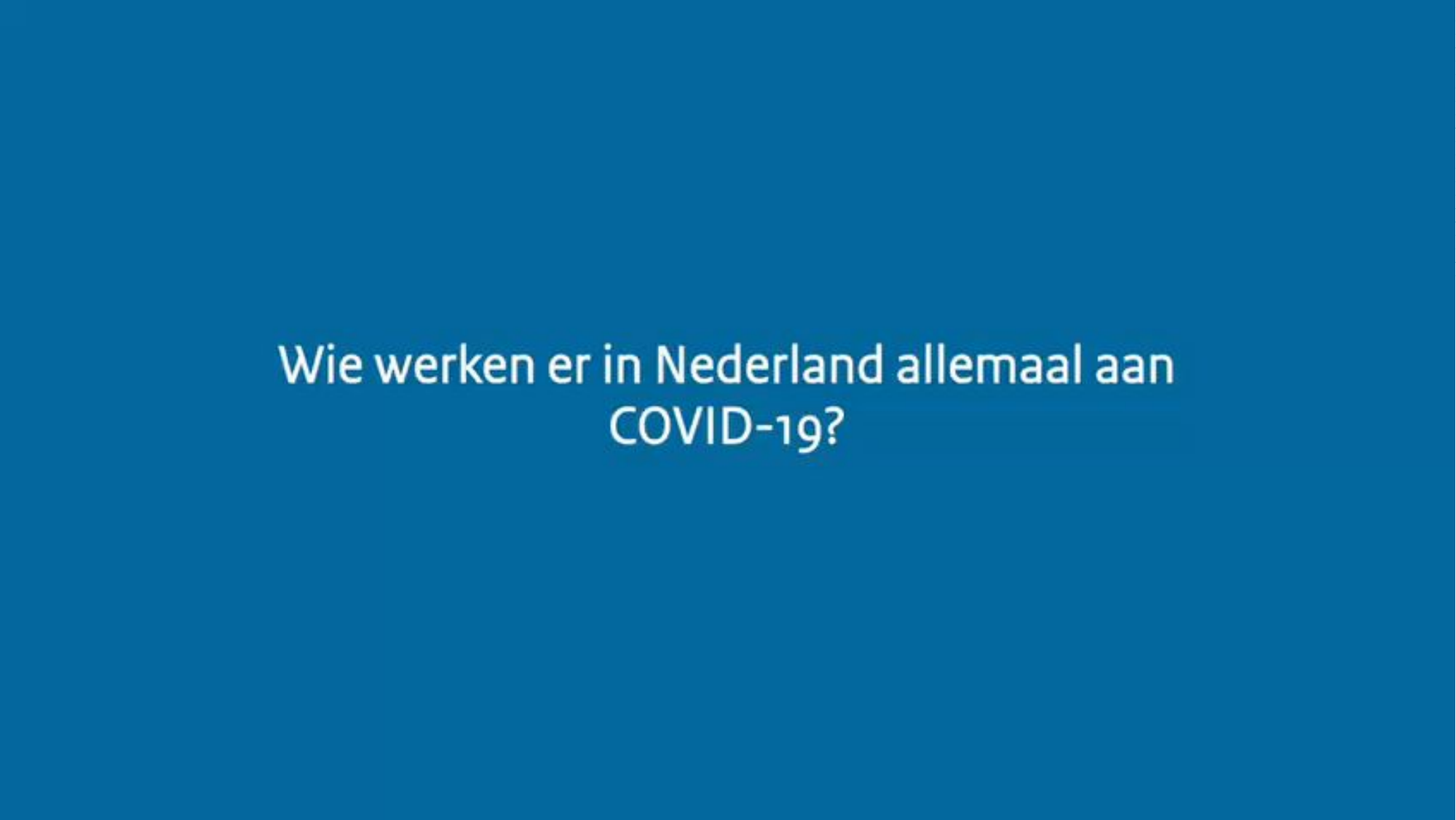

#
1

## Slide 2
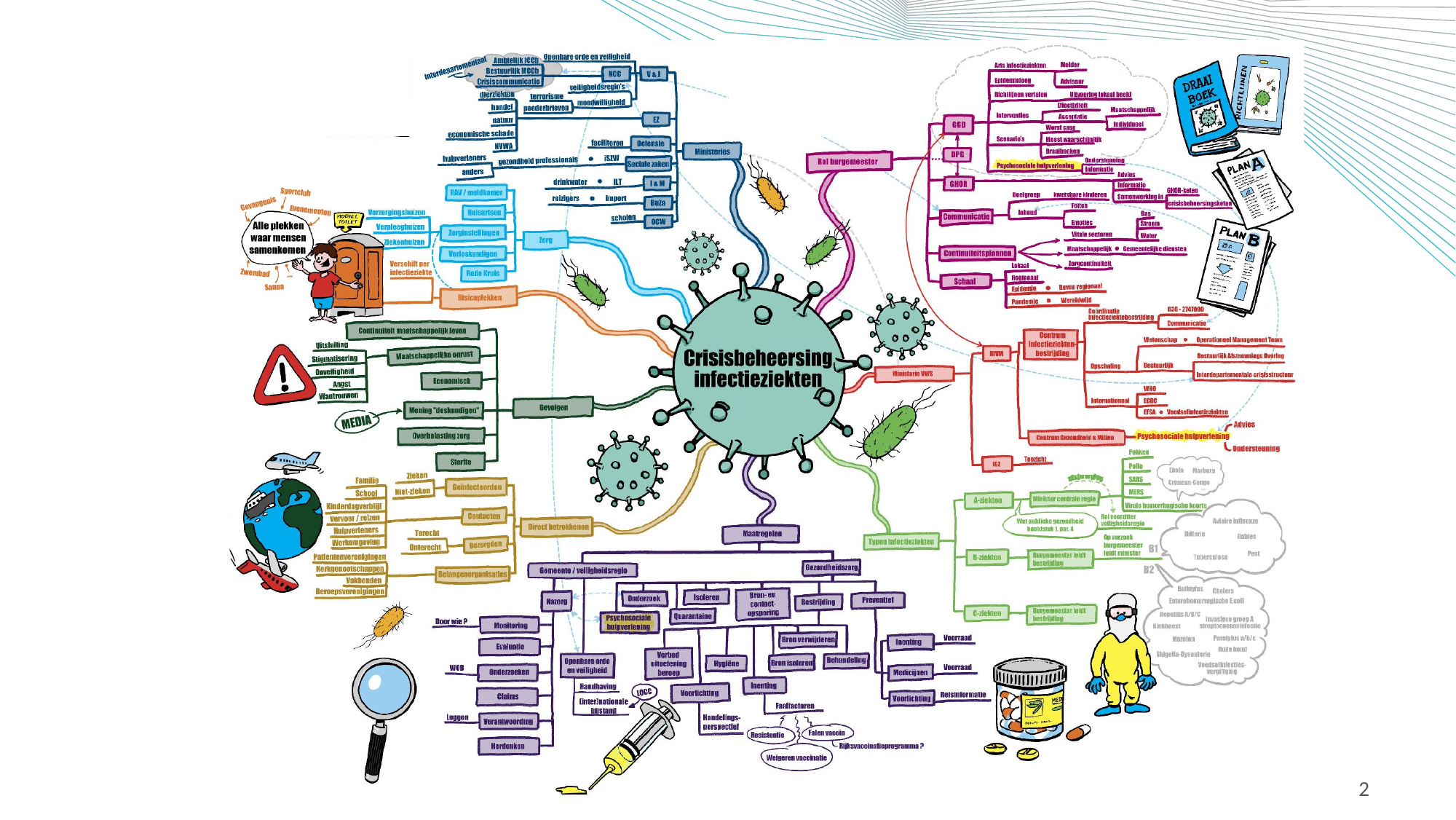

#
2

Supplement: Supplementary file 3 — Supporting information. [file HEX-27-e14170-s003.pptx]
